# Crystal growth, layered structure and luminescence properties of K_2_Eu(PO_4_)(WO_4_)†

**DOI:** 10.1039/d2ra00932c

**Published:** 2022-03-22

**Authors:** Kateryna V. Terebilenko, Vitalii P. Chornii, Valeriіa O. Zozulia, Il'ya A. Gural'skiy, Sergiu G. Shova, Serhii G. Nedilko, Mykola S. Slobodyanik

**Affiliations:** Taras Shevchenko National University of Kyiv Volodymyrska St. 64 Kyiv 01601 Ukraine kterebilenko@gmail.com; National University of Life and Environmental Sciences of Ukraine Heroiv Oborony St, 15 Kyiv 03041 Ukraine; “Petru Poni” Institute of Macromolecular Chemistry 41A Aleea Gr. Ghica Voda 700487 Iasi Romania

## Abstract

K_2_Eu(PO_4_)(WO_4_) has been prepared *via* the high-temperature solution growth (HTSG) method using K_2_WO_4_–KPO_3_ molten salts as a self-flux and characterized by single-crystal X-ray diffraction analysis, IR and luminescence spectroscopy. The structure of this new compound features a 2D framework containing [EuPO_6_]^4−^ layers, which are composed of zigzag chains of [EuO_8_]_n_ interlinked by slightly distorted PO_4_ tetrahedra. Isolated WO_4_ tetrahedra are attached above and below these layers, leaving space for the K^+^ counter-cations. The photoluminescence (PL) characteristics (spectra, line intensity distribution and decay kinetics) confirm structural data concerning one distinct position for europium ions. The luminescence color coordinates suggest K_2_Eu(PO_4_)(WO_4_) as an efficient red phosphor for lighting applications.

## Abstract 2

K_2_Eu(PO_4_)(WO_4_) has been prepared *via* the high-temperature solution growth (HTSG) method using K_2_WO_4_–KPO_3_ molten salts as a self-flux and characterized by single-crystal X-ray diffraction analysis, IR and luminescence spectroscopy.

## Graphical abstract

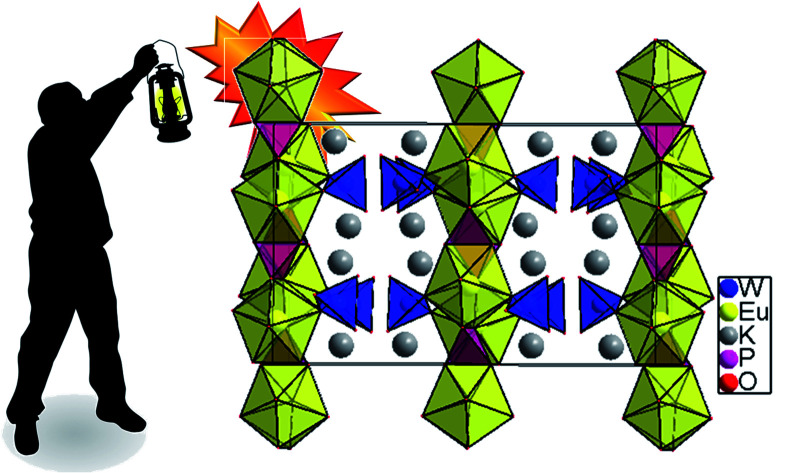

## Introduction

Lanthanide-containing complex oxides based on phosphate, vanadate, molybdate and tungstate have been actively studied as phosphors for solid lighting technologies, particularly for white light emitting diodes (LEDs).^1–3^ Among them, much attention has been paid to Eu^3+^-containing compounds due to their prominent photoluminescence (PL) in the red spectral region. This photoluminescence can be excited by (1) a direct excitation of Eu^3+^ ions through intraconfigurational 4f^6^–4f^6^ absorption transitions, (2) charge transfer transitions from ligand to europium(iii) ion or (3) an energy transfer of absorbed energy from a host to rare-earth (RE) ions. Among these ways the 4f^6^–4f^6^ transitions lead to low values of absorption cross-sections because they are forbidden from the viewpoint of quantum mechanics. The wide band of O^2−^ → Eu^3+^ charge–transfer transition has high intensity and is located at ∼225–300 nm for many oxide compounds.^4–7^ However, absence of cheap semiconductor chips with intensive radiation at this short wavelength makes excitation through O^2−^ → Eu^3+^ mechanism inconvenient for LED applications. The third takes place through absorption of light by structural moieties of the host with further transfer of absorbed energy to Eu^3+^-based emission centres. In case of molybdate and tungstate compounds this type of absorption is realized through O^2−^ → Mo^6+^ or O^2−^ → W^6+^ charge transfer providing a wide band in 250–350 nm range in the PL excitation spectra.^7–10^ From LED application viewpoint suitable PL excitation can be achieved simultaneously through a direct f–f transition in rare earth ions and by light absorption of the host. It is worth noting that the most intensive absorption usually takes place in the energy region near to the host band gap. In case of complex oxides with molecular anions listed above, the typical band gap values fall within 3–5 eV energy region that is considered as the most convenient for phosphor elaboration.^1^

One of the advantages of molybdate and tungstate hosts for rare earth ions is related with weak concentration quenching of luminescence caused by these ions, particularly Eu^3+^ ones. This phenomenon is explained by quite inefficient energy transfer between Eu^3+^ ions those ones located at the distances at about 4–5 Å each from another.^8,11^ Some of the structures discussed are layered and characterized by preferable directions for energy transfer. Layered crystal structure is inherent also to mixed-anion compounds with general formula A_2_R(PO_4_)(MO_4_), where A = Na or K; R = Y, Bi or RE; M = Mo or W.^12–18^ Although the first structure of this family, Na_2_Y(PO_4_)(MoO_4_), was reported more than three decades ago,^12^ there are some gaps in the studies of layered phosphomolybdates concerning both crystal structure and their physicochemical properties. To the best of our knowledge there are no reports in the literature on synthesis, crystal structure and optical properties of K_2_Eu(PO_4_)(WO_4_). Importantly, an isostructural compound K_2_Eu(PO_4_)(MoO_4_) has been reported as an efficient phosphor possessing intensive red luminescence.^15^ The luminescence properties of the mentioned above phosphomolybdate^15^ has been studied in a light of bismuth by europium substitution in the K_2_Bi(PO_4_)(MoO_4_) structure.^19^ The further studies of K_2_Eu(PO_4_)(MoO_4_) luminescence have shown that its quantum yield is close to 96% and 86% when the PL excitation is performed at 394 and 465 nm, respectively.^20^ It is worth noting, the substitution of molybdenum by tungsten in K_2_Bi(PO_4_)(MoO_4_) : Eu phosphor improves intensity of luminescence with best results achieved for K_2_Bi(PO_4_)(WO_4_) : 0.8Eu.^19^

The effect of anion substitution in similar layered compounds has been shown to be a driving force in separating emission centers and therefore enhancing the thermal stability and increasing the critical concentration of activator ions.^21,22^ Thus, anionic ratio MoO_4_^2−^/PO_4_^3−^ for Na_2−*n*_Y(MoO_4_)_1+*n*_(PO_4_)_1−*n*_ : Tb^3+^,Eu^3+^ has been used for improving the thermal stability of phosphors obtained.^22^ In this light one should admit significant difference in excitation and luminescence spectra for isostructural hosts containing molybdate^15,21,23^ and tungstate groups.^24,25^ To clarify this phenomenon more spectral data for phosphotungstates should be collected and analysed.

In the present paper we report single crystal growth, crystal structure and luminescence properties of the layered phosphor K_2_Eu(PO_4_)(WO_4_).

## Experimental

### Synthetic procedures

Single crystals of K_2_Eu(PO_4_)(WO_4_) have been grown by the high-temperature solution growth method from K_2_WO_4_ : KH_2_PO_4_ : Eu_2_O_3_ molten mixture in the ratio 4.99 : 4.99 : 0.02. The precursors without further purification have been mixed and grinded together in an agate mortar and melted in a platinum crucible at 1100 °C. On the next stage the high-temperature solution prepared has been kept at this temperature for 2 hours under stirring in order to reach the homogeneity. The molten mixture has been cooled down to 750 °C at the rate of 80 °C h^−1^. At this stage the melt has been poured down on a copper sheet and a crystalline product has been left in the furnace for slow cooling to room temperature. The colourless plates have been leached out with hot deionized water and characterized by IR and single crystal X-ray diffraction.

### Crystallography

A suitable single crystal of K_2_Eu(PO_4_)(WO_4_) was selected and mounted on an Xcalibur, Eos diffractometer (Mo-K_α_ radiation, *λ* = 0.71073). The crystal was kept at 293 K during data collection. Using Olex2,^26^ the structure was solved with the SHELXT^27^ structure solution program (Intrinsic Phasing method) and refined with the SHELXL^28^ refinement package (Least Squares minimisation). Crystallographic data and structure refinement parameters for K_2_Eu(PO_4_)(WO_4_) are summarized in Table 1.

**Table 1:** Crystal data and structure refinements for K_2_Eu(PO_4_)(WO_4_)

Empirical formula	EuK_2_O_8_PW
Formula weight	572.98
Temperature/K	293(2)
Crystal system	Orthorhombic
Space group	*Ibca*
*a*/Å	6.9856(4)
*b* Å	12.2954(5)
*c*/Å	19.7434(9)
Volume/Å^3^	1695.79(15)
*Z*	8
*ρ*_calc,_ g cm^−3^	4.489
μ mm^−1^	22.064
Crystal size/mm^3^	0.25 × 0.25 × 0.02
2*Θ* range for data collection/°	4.126 to 58.916
Reflections collected	5948
Independent reflections	1049 [*R*_int_ = 0.0528, *R*_sigma_ = 0.0374]
Data/restraints/parameters	1049/0/61
Goodness-of-fit on F^2^	1.084
Final *R* indexes [*I*>=2*σ* (*I*)]	*R*_1_ = 0.0326, *wR*_2_ = 0.0799

Further details on the structure refinements of K_2_Eu(PO_4_)(WO_4_) may be obtained from the Fachinformationszentrum Karlsruhe, D-76344 Eggenstein-Leopoldshafen (Germany), by quoting the Registry no. CSD – 2151141.

### Sample characterization

Investigations of the thermal behavior of the K_2_Eu(PO_4_)(WO_4_) have been performed using a Shimadzu DTG-60H simultaneous thermogravimetry/differential thermal analyzer. The sample and the reference (α-Al_2_O_3_) were heated up to 900 °C in Pt crucibles under an air atmosphere at 10 °C min^−1^.

IR spectrum has been measured on a PerkinElmer Spectrum BX FTIR spectrometer in the frequency range 400–4400 cm^−1^ in KBr pellets.

The PL emission and excitation spectra of the samples have been recorded at room temperature using a DFS-12 spectrometer equipped with a FEU-79 photomultiplier. A powerful Xenon arc lamp (DXeL-1000) combined with a DMR-4 prism monochromator was used as source of the excitation light. All the spectra have been corrected on system response.

The PL kinetics have been measured with use of a MSA-300 multiscaler photon counter and a blue LED (*λ*_rad_ = 465 nm) operating at pulse regimes as a source of the PL excitation.

## Results and discussion

### Crystal structure

**Fig. 1:**
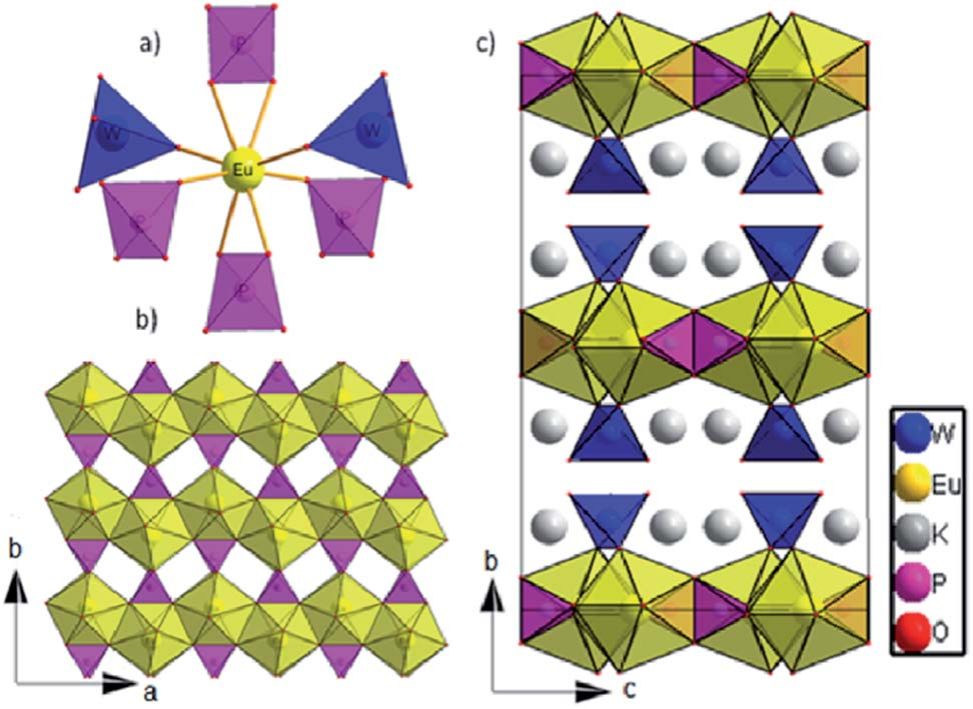
(a) The nearest surrounding of europium cation in K_2_Eu(PO_4_)(WO_4_) structure; (b) 2D layer at ab plane; c) The crystal structure view along *a* axis.

The dipotassium europium(iii) phosphate(V) tungstate(VI) K_2_Eu(PO_4_)(WO_4_) crystallizes in the *Ibca* space group (orthorhombic crystal system) with eight K_2_Eu(PO_4_)(WO_4_) formula units per unit cell (Table 1). There is one crystallographically unique europium cite in the Wyckoff special position 8d, showing a coordination sphere of eight oxygen atoms in the shape of a triangular dodecahedron (Fig. 1a).

The distortions of the coordination environment of europium, phosphorus and tungsten have been calculated with Shape 2.0 program^29^*via* the Continuous Shape Measure method. The value of *S* = 2.908 was obtained for the Eu environment, which means a quite essential deviation from the ideal triangular dodecahedron.^30^ Each Eu cation is surrounded by two tungstate and four phosphate groups; two of them are coordinated in a bidentate manner (Fig. 1a). Among Eu–O bond distances, those that correspond to bidentately – coordinated phosphate groups are the largest (2.433(6) Å and 2.475(5) Å, respectively, see Table 2). Thus, K_2_Eu(PO_4_)(WO_4_) comprises non-condensed phosphate and tungstate tetrahedra.

**Table 2:** Parameters of oxygen polyhedra in K_2_Eu(PO_4_)(WO_4_)

Moieties (MO_*x*_)	EuO_8_	KO_8_	PO_4_	WO_4_
Bond lengths (Å)	2.327(6) ×2	2.659(6)	1.515(5)×2	1.763(6)×2
	2.392(6) ×2	2.705(6)	1.543(6)×2	1.788(6)×2
	2.433(6) ×2	2.782(6)		
	2.475(5) ×2	2.970(7)		
		2.980(7)		
		3.069(6)		
		3.186(7)		
		3.198(7)		
*M* Site symmetry	*C*_2_	*C*_1_	*C*_2_	
Polyhedron type	Triangular	Biaugmented trigonal prism	Tetrahedron	
	Dodecahedron			
Symmetry of ideal polyhedron	*D*_2d_	*C*_2v_	*T*_d_	
*S*	2.908	4.036	0.212	0.047

Each tetrahedrally coordinated phosphorus(v) and tungsten(vi) atoms are crystallographically unique and are located at the Wyckoff positions 8d and 8e, respectively. They are surrounded by four oxygen atoms forming bisphenoidally distorted tetrahedra. The small values of the *S* parameter (Table 2) indicate a slight deviation from ideal tetrahedra for both PO_4_ and WO_4_. Despite the fact that both tetrahedral moieties exhibit *C*_2_ site symmetry, phosphate tetrahedra are found to be more disported than tungstate ones.

**Fig. 2:**
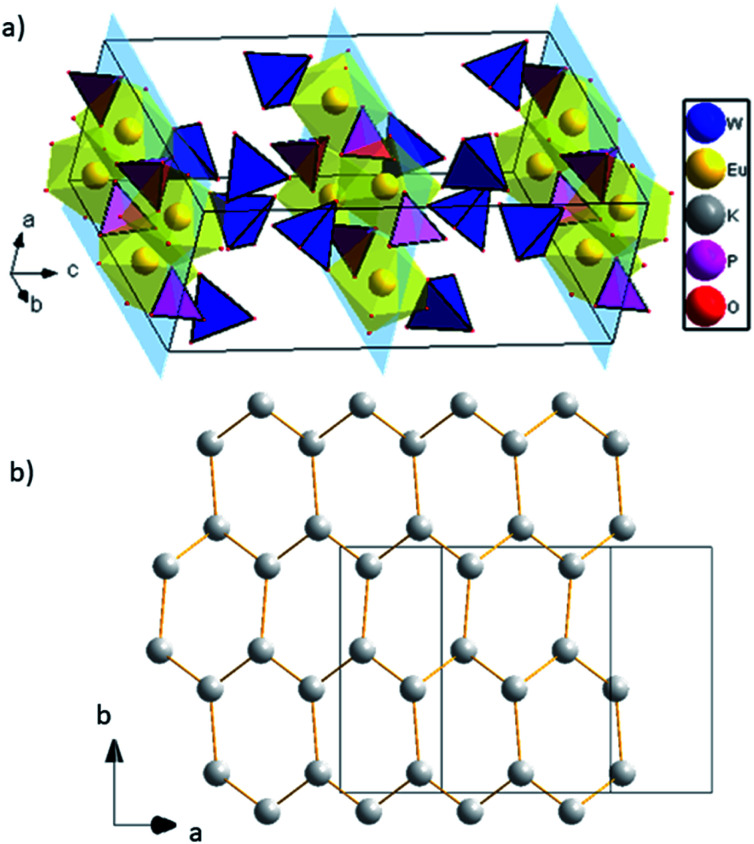
(a) 2D layers [EuPO_6_]^4-^, K atoms are omitted for clarity; (b) graphene-like layer of K cations.

Europium triangular dodecahedra are connected by common edges forming a zigzag chain along *a* axis (Fig. 1b). These [EuO_8_]_n_ zig-zag chains are linked by phosphate tetrahedra building a layer in ab-plane (Fig. 1b). Finally, the WO_4_ tetrahedra are attached to the plane from both sides along *b* axis (Fig. 1c). Layers [EuPO_6_]^4-^ represent the nearest Eu⋯Eu contacts 3.9644(4) Å, while the other ones are much longer and belong to different layers (Fig. 2a). In comparison to K_2_Eu(PO_4_)(MoO_4_) structure^15^ the shortest distance between neighbor Eu⋯Eu contacts are much shorter being 3.5 Å. Potassium cations are found on crystallographically unique 16f Wyckoff positions, showing a coordination sphere of eight oxygen atoms in the shape of biaugmented trigonal prism (Table 2), which reside among the voids between the neighboring sheets.

Interestingly, potassium cations within the layer form a graphene-like sheets along direction (1 0 1) with the shortest K to K distance equal to 3.9869(1) Å, and the longest are 4.2492(1) Å (Fig. 2b).

### Thermal analysis

K_2_Eu(PO_4_)(WO_4_) is characterized by high thermal stability in the temperature range of 20–900 °C with slight weight loss less than 0.65% most probably explained by adsorbed water. Taking into consideration that melting point of K_2_Eu(PO_4_)(MoO_4_)^15^ is above 1050 °C, the thermal stability of the tungstate-containing analogue may be expected to be higher.

### IR spectroscopy

**Fig. 3:**
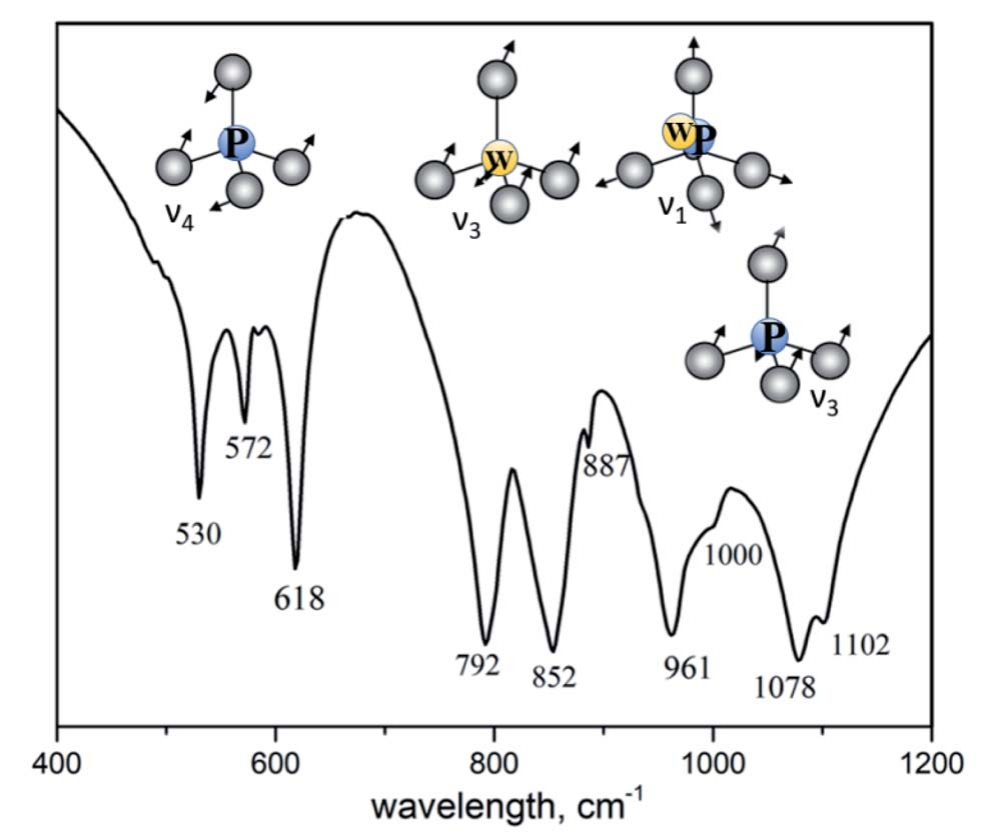
IR spectrum of K_2_Eu(PO_4_)(WO_4_).

Fig. 3 shows IR spectrum of K_2_Eu(PO_4_)(WO_4_) in the region of 400–1200 cm^−1^ where the most intensive absorption bands are located. From structural point of view, the titled compound is isostructural to the parent one K_2_Bi(PO_4_)(MoO_4_).^14^ Spectroscopic data illustrates the difference in the local symmetry of tetrahedral units. Thus, the wide band located at 1078 cm^−1^ with a shoulder at 1102 cm^−1^ is ascribed to the asymmetric stretching vibration *ν*_3_(F_2_) in PO_4_ tetrahedra.^31^ On the contrary, this band in IR spectrum of K_2_Bi(PO_4_)(MoO_4_) is redshifted toward 1055 cm^−1^. The same situation is found for a band at 961 cm^−1^ with a shoulder at 1000 cm^−1^ which is ascribed to the symmetric stretching vibration *ν*_1_(F_2_) in PO_4_ tetrahedra. The corresponding band is found at 945 cm^−1^ in the case of K_2_Bi(PO_4_)(MoO_4_) (Table 3). The bands in a range of 887–792 cm^−1^ can be attributed to stretching vibrations in the WO_4_ tetrahedra. The 500–650 cm^−1^ region shows three bonds expected for *ν*_4_(F_2_) of PO_4_ tetrahedra bending vibrations: 618, 572 and 530 cm^−1^. The characteristic bands observed for the title compound agree well with other isostructural compounds (Table 3).

**Table 3:** IR wavenumbers (cm^−1^) and band assignments for A_2_R(PO_4_)(MO_4_) (A–K, Rb, R–Bi, Eu, M−Mo, W)

Host	*ν*_3_(PO_4_)	*ν*_1_(PO_4_)+ *ν*_1_(MO_4_)	*ν*_3_(MO_4_)	*ν*_4_(PO_4_)
K_2_Eu(PO_4_)(WO_4_)	1102	1000	887	618
	1078	961	852	572
			792	530
Na_2_Y(PO_4_)(WO_4_)^32^	1095	985	860	620
		945	823	575
			797	535
Rb_2_Eu(PO_4_)(MoO_4_)^33^	1075	950	901	608
			825	562
			780	524
K_2_Gd(PO_4_)(WO_4_)^34^	1081	962	843	—
			786	
K_2_Bi(PO_4_)(MoO_4_)^14^	1055	945	895	590
			860	555
			815	520
			790	
			740	

### Luminescence spectroscopy

The phosphotungstate K_2_Eu(PO_4_)(WO_4_) reveals intensive red photoluminescence in case of excitation under UV and blue light excitation at room temperature. The corresponding spectra consist of relatively narrow emission bands which are related with ^5^D_0_ → ^7^F_*J*=1−4_ electronic transitions in Eu^3+^ ions (Fig. 3). The most intensive bands located near 615 and 700 nm correspond to forced electric dipole transitions ^5^D_0_ → ^7^F_2_ and ^5^D_0_ → ^7^F_4_, respectively. It should be pointed out that intensity of the bands of ^5^D_0_ → ^7^F_4_ transitions is abnormally high in respect to ^5^D_0_ → ^7^F_1_ ones for all spectra obtained. This phenomenon is not typical for Eu^3+^ ions emission in solids but it has been observed earlier for some hosts, in particular phosphate and tungstate ones.^35–38^ High intensity of the ^5^D_0_ → ^7^F_4_ transitions was explained earlier assuming the highly polarizable chemical environment for Eu^3+^ emission centre.^32^ In our case, the europium cations are surrounded by 4 phosphate and 2 tungstate tetrahedra (Fig. 1a). Taking into consideration the second coordination sphere of EuO_8_ polyhedra one can admit more covalent character in case of P–O–Eu bonds in comparison to W–O–Eu ones. These structure-related peculiarities can be regarded as the source of higher polarizability of Eu^3+^ environment in the K_2_Eu(PO_4_)(WO_4_) and, consequently, the reason for high emission intensity of the ^5^D_0_ → ^7^F_4_ transitions.

Moreover, the ratios between the PL intensities of ^5^D_0_ → ^7^F_4_ and ^5^D_0_ → ^7^F_*J*=1,2_ transitions depend on excitation wavelength (calculated values are collected in the Table 4). This phenomenon can be explained by the influence of electron-phonon coupling in two types of luminescence centers: a regular EuO_8_ polyhedron and a defect-containing one. The high value of the asymmetry ratio, *R* = *I*(^5^D_0_ → ^7^F_2_)/*I*(^5^D_0_ → ^7^F_1_), in the Table 4 indicates that Eu^3+^ cations are located at low-symmetry sites without inversion centre in accordance with structural data. Ratio of intensities *I*(^5^D_0_ → ^7^F_4_)/*I*(^5^D_0_ → ^7^F_2_) changes slightly when *λ*_ex_ is switched from 380 to 393 nm that is also related with the impact of defect-containing luminescence centers.

**Table 4:** Ratios between total^a^ intensities of ^5^D_0_ → ^7^F_*J*_ transitions in Eu^3+^ ions in K_2_Eu(PO_4_)(WO_4_) and chromaticity coordinates

*λ*_ex_, nm	*R* = *I*(^7^F_2_)/*I*(^7^F_1_)	*I*(^7^F_4_)/*I*(^7^F_1_)	*I*(^7^F_4_)/*I*(^7^F_2_)	*x*	*y*
380	3.05	4.26	1.40	0.647	0.349
393	3.26	5.40	1.66	0.652	0.347
466	2.96	4.94	1.67	0.647	0.353

a total intensity is calculated as area under spectra in the regions 580–600 (^5^D_0_ → ^7^F_1_), 600–630 (^5^D_0_ → ^7^F_2_) and 680–710 nm (^5^D_0_ → ^7^F_4_).

**Fig. 4:**
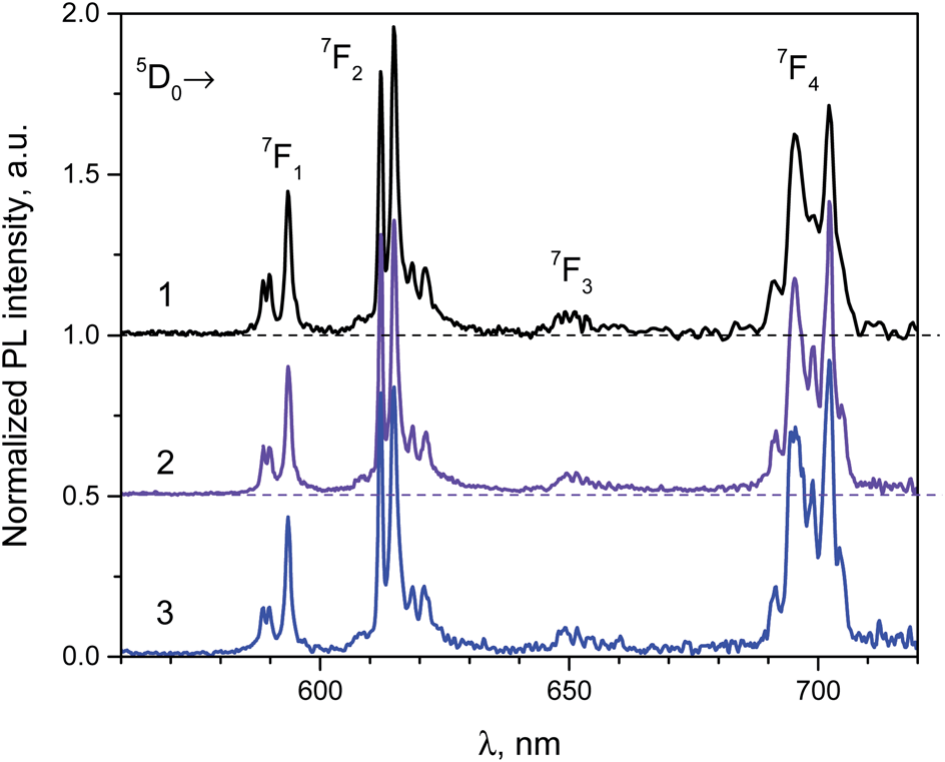
Luminescence spectra of the K_2_Eu(PO_4_)(WO_4_) obtained for excitation at *λ*_e*x*_ = 380 (1), 393 (2) and 466 nm (2) at room temperature.

The normalized PL excitation spectra of the Eu^3+^-related luminescence in the K_2_Eu(PO_4_)(WO_4_) are shown in Fig. 4. The most intensive band peaking at 393 nm in the spectra is related with ^7^F_0_ → ^5^L_6_ transition. Less intensive bands are located near 319 (^7^F_0_ → ^5^H_*J*_), 360 (^7^F_0_ → ^5^D_4_), 375 (^7^F_0_ → ^5^G_*J*_), 380 (^7^F_0_ → ^5^L_7,8_), 415 (^7^F_0_ → ^5^D_3_), 465 (^7^F_0_ → ^5^D_2_), 534 and 543 nm (^7^F_0-2_→^5^D_1_). The wide band with maximum below 260 nm is ascribed to O^2−^ → Eu^3+^ charge transfer that typically observed for Eu^3+^-containing oxide compounds, *e.g.* in case of the K_2_Eu(PO_4_)(MoO_4_) ones.^19,20^

**Fig. 5:**
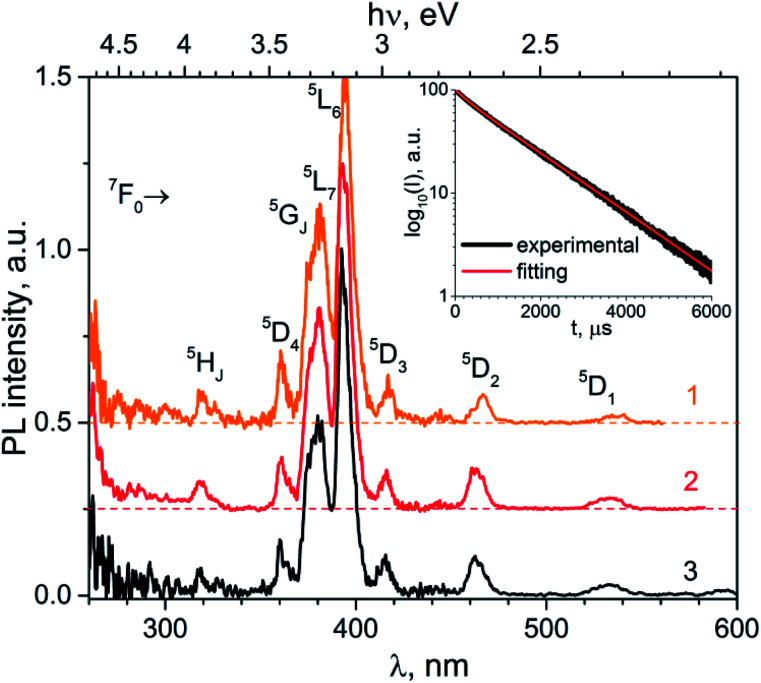
PL excitation spectra of the K_2_Eu(PO_4_)(WO_4_) registered at *λ*_em_ = 594 (1), 615 (2) and 702.5 nm (3); *T* = 300 K. Inset: PL decay curve in semi-logarithmic scale (*λ*_ex_ = 465 nm, *λ*_em_ = 615 nm) and its fitting with formulae *I* = *I*_1_ × exp(−*t*/*τ*_1_) + *I*_2_ × exp(−*t*/*τ*_2_).

Minor changes in the PL excitation spectra can be seen in the regions of ^7^F_0_ → ^5^L_6_ and ^7^F_0_ → ^5^D_2_ electronic transitions. In case of registration at *λ*_em_ = 594 nm the band maxima of these transitions are shifted toward longer wavelength in respect to corresponding bands in the PL excitation spectra registered at *λ*_em_ = 615 and 702.5 nm. These shifts are about 0.02 eV in energy scale that is comparable with *kT* value at room temperature (0.026 eV). It has been found that under excitation at 465 nm and registration at 615 nm the PL kinetics curve can be fitted by double exponential decay: *I* = 10.6 × exp(−*t*/*τ*_1_) + 88.4 × exp(−*t*/*t*_2_) with time constants *τ*_1_ = 277 ± 5 μs and *τ*_2_ = 1527 ± 2 μs. Average lifetime for K_2_Eu(PO_4_)(WO_4_) has a value of 1379 μs when calculated with formula *τ*_avg_ = (*I*_1_ × *τ*_1_ + *I*_2_ × *τ*_2_)/(*I*_1_ + *I*_2_). This value is higher than common ones for tungstate-containing compounds, namely, 498 μs found for KEu(WO_4_)_2_.^34^ The increased PL lifetimes might be related to the charge transfer band lying at higher energies as it has been found for isostructural compound, K_2_Eu(PO_4_)(MoO_4_) with PL emission component having *τ* ≈ 2050 μs at room temperature^20^ when *λ*_ex_ = 465 nm and *λ*_em_ = 615 nm.Fig. 5 Significant difference in average lifetime for tungstate and molybdate-containing isostructural compounds may be also related with different energies of charge transfer bands.^20^

Luminescence data are found to be in agreement with structural peculiarities of the K_2_Eu(PO_4_)(WO_4_). The spectroscopic characteristics can be discussed in a light of one unique Eu position in a quite distorted eight-fold coordination and the arrangement of these polyhedra into 2D layers. At the same time, the presence of peak positions' shifts in the PL excitation spectra, complex dependence of the asymmetry ratio *R* on excitation wavelength, and two components observed in kinetics of the PL decay cannot be omitted and requires additional study. Similar situation has been observed for other Eu-containing compounds where defects in oxygen environment of europium^35–40^ caused by annealing during synthetic procedure leads to distinguish two types of luminescence centers. The first one is associated with Eu in regular EuO_8_ dodecahedra, while the second center may be related with oxygen vacancies.^37,41^ The latter assumption is supported with previously reported data for oxide phosphors containing phosphate and tungstate groups.^42^ Moreover, the complex nature of the PL decay, which is a superposition of fast and slow components is also more likely to be defect-related. Thus, the contribution of the fast component to the emission (*I*_1_·*τ*_1_ ≈ 2900 r.u.), which is related with vacancy-containing centers is ∼45 times smaller than the slow component one (*I*_2_·*τ*_2_ ≈ 135 000 r.u.).

The further studies of the PL properties especially at low temperatures are very necessary for clarifying noted assumption.

Due to intensive red luminescence of phosphotungstate K_2_Eu(PO_4_)(WO_4_) can be considered as a suitable phosphor for luminescent lighting application. The calculated values of chromaticity coordinates (x y) are collected in Table 4. The colour coordinates are close to those of the NTSC standard for red colour (0.67; 0.33) for all PL excitation studied. High intensity of ^5^D_0_ → ^7^F_2_ observed for K_2_Eu(PO_4_)(WO_4_) can be considered for applications as luminescent down-shifting for white light emitting diodes.

## Conclusions

In summary, K_2_Eu(PO_4_)(WO_4_) crystals have been successfully prepared by the high-temperature solution growth, and its structure, thermal stability, chromaticity coordinates, luminescence spectra and decay kinetics have been investigated in detail. The crystal structure contains non-condensed phosphate and tungstate tetrahedra interlinked with condensed in a zigzag chain EuO_8_ polyhedra, while potassium cations reside in interlayer space. The phosphor can be efficiently excited by light at the 360–480 nm spectral region and give rise to bright luminescence with the most intensive bands located near 615 and 700 nm correspond to ^5^D_0_ → ^7^F_2_ and ^5^D_0_ → ^7^F_4_ forced electric dipole transitions in europium cation, respectively. The abnormally high intensity of ^5^D_0_ → ^7^F_4_ transitions is ascribed to polarizability of Eu^3+^ environment in the K_2_Eu(PO_4_)(WO_4_). Luminescent properties are consistent with the structural characteristics of the studied crystals. In particular, the PL results confirm that all positions of europium ions in the crystal lattice are equivalent. The photoluminescence characteristics obtained indicate that titled compound has a potential application as a red-light emitting phosphor.

## Conflicts of interest

There are no conflicts to declare.

## Supplementary Material

RA-012-D2RA00932C-s001
